# Conditional and Unconditional Tests (and Sample Size) Based on Multiple Comparisons for Stratified 2 × 2 Tables

**DOI:** 10.1155/2015/147038

**Published:** 2015-05-14

**Authors:** A. Martín Andrés, I. Herranz Tejedor, M. Álvarez Hernández

**Affiliations:** ^1^Bioestadística, Facultad de Medicina, University of Granada, 18071 Granada, Spain; ^2^Bioestadística, Facultad de Medicina, University Complutense of Madrid, 28040 Madrid, Spain; ^3^Departamento de Estadística e Investigación Operativa, University of Vigo, 36310 Vigo, Spain

## Abstract

The Mantel-Haenszel test is the most frequent asymptotic test used for analyzing stratified 2 × 2 tables. Its exact alternative is the test of Birch, which has recently been reconsidered by Jung. Both tests have a conditional origin: Pearson's chi-squared test and Fisher's exact test, respectively. But both tests have the same drawback that the result of global test (the stratified test) may not be compatible with the result of individual tests (the test for each stratum). In this paper, we propose to carry out the global test using a multiple comparisons method (MC method) which does not have this disadvantage. By refining the method (MCB method) an alternative to the Mantel-Haenszel and Birch tests may be obtained. The new MC and MCB methods have the advantage that they may be applied from an unconditional view, a methodology which until now has not been applied to this problem. We also propose some sample size calculation methods.

## 1. Introduction

In statistics it is very usual to have to verify whether association exists between two dichotomic qualities. This is especially frequent in medicine, for example, where the aim is to assess whether the presence or absence of a risk factor conditions the presence or absence of a disease or compare two treatments whose answers are success or failure, and so forth. In all the cases the problem produces data whose frequencies are presented in a 2 × 2 table: the two levels of one of the qualities are set out in the rows, the two levels of the other quality in the columns, and the observed frequencies are set out inside the table.

The exact and the asymptotic analyses of a 2 × 2 table have their roots in the origins of statistics, and hundred of papers have been devoted to the problem [1]. It is traditional to carry out the exact independence test using the Fisher exact test, which is a conditional test (because it assumes that the marginals of the rows and columns are previously fixed). More than thirty years has passed since the situation changed, and it is well known that the unconditional exact test tends to be less conservative and more powerful than the conditional test [2–4], because the loss of information as a result of conditioning may be as high as 26% [5]. The unconditional tests assume that it is only the values that were really previously fixed: the marginal of the rows, the marginal of the columns or the total data in the table. This causes two types of unconditional test: that of the double binominal model (the first two cases) and that of the multinomial model (the third case). The same can be said of the asymptotic tests, generally based on Pearson's chi-squared statistic with different corrections for continuity (cc). However, the unconditional exact tests have the great disadvantage of being very laborious to compute. An overall view of the problem can be seen in Martín Andrés [1, 6].

Frequently the individuals who take part in the study are stratified in groups based on a covariate such as sex or age, which gives rise to several 2 × 2 tables. In this case the aim is to contrast the independence of both the original dichotomic qualities, bearing in mind the heterogeneity of the populations defined by the strata. To this end, the most frequent approach is to suggest a test under the null hypothesis of Mantel-Haenszel for which the odds ratio (or the risk ratio) for all the strata is equal to unity. For this purpose the most frequent asymptotic tests are those of Cochran [7] and Mantel and Haenszel [8], both of which are very similar; the exact version of the test is due to Birch [9] (and has recently been reconsidered by [10]). In all these cases the proposed tests are conditional and, when there is only one stratum, the test for the case of only one 2 × 2 table is obtained (Fisher's exact test or Pearson's chi-squared test). Moreover, Jung [10] and Jung et al. [11] propose a sample size calculation method, asymptotic in the first and exact in the second.

The procedures indicated have the drawback of almost all the tests for a global null hypothesis like the one in question that the result of the global (stratified) test may not be compatible with that of the individual tests (the test for each stratum). In this paper, we propose a global test (MC test) which does not have this disadvantage because it is based on a multiple comparisons method: the global test is significant if and only if at least one of the individual tests is significant. In return the MC test will have the drawback of being less powerful, given that it must control both the alpha error of the global test and the alpha errors in the individual tests. Because of this, another procedure is proposed (MCB test) which only controls the alpha error of the global test (just as in the classic stratified tests), although the alpha error in the individual tests will only exceed the nominal value on a few occasions (and generally by very little). The two procedures are applicable from both the conditional and the unconditional point of view and also when carrying out an asymptotic test or an exact test. The advantage of applying them in the form of an unconditional test is that in this way the loss of power mentioned above is reduced with regard to the classic global tests. In addition this paper shows that the asymptotic tests function well, even for small samples, if they are carried out with the appropriate continuity correction. And finally, the sample size for almost all the cases studied (exact or asymptotic tests, conditional or unconditional tests) is determined.

## 2. Hypothesis Test

### 2.1. Notation, Models, and Example

In the following (without loss of generality) it will be assumed that each 2 × 2 table refers to the successes or failures in two treatments which are applied to *m*_*j*_ and *n*_*j*_ individuals, respectively. Let *J* be the number of strata, *N*_*j*_ = *m*_*j*_ + *n*_*j*_ (*j* = 1,…, *J*) the total of individuals in the stratum *j*, *N* = ∑_*j*_ *N*_*j*_ the total sample size, $\left\{x_{j},{\overset{-}{x}}_{j}=m_{j}-x_{j}\right\}$ and $\left\{y_{j},{\overset{-}{y}}_{j}=n_{j}-y_{j}\right\}$ the number of successes and the number of failures with the treatments 1 and 2, respectively, and *z*_*j*_ = *x*_*j*_ + *y*_*j*_ and ${\overset{-}{z}}_{j}={\overset{-}{x}}_{j}+{\overset{-}{y}}_{j}$ the total number of successes and failures in the stratum *j* respectively. These data may be summarized as shown in Table 1. Once the experiment has been performed, the values obtained will be written with an extra subindex “0,” that is, *x*_*j*0_, *y*_*j*0_, *m*_*j*0_, *N*_*j*0_,….

Let *p*_*j*_ and *q*_*j*_ (${\overset{-}{p}}_{j}=1-p_{j}$ and ${\overset{-}{q}}_{j}=1-q_{j}$) be the probabilities of success (failure) with treatments 1 and 2 in the stratum *j*, respectively. The odds ratio for each stratum is $\theta_{j}=p_{j}{\overset{-}{q}}_{j}/{\overset{-}{p}}_{j}q_{j}$, and the aim is to contrast the null hypothesis *H*: *θ*_1_ = ⋯ = *θ*_*J*_ = 1 against an alternative hypothesis with one tail (*K*: *θ*_*j*_ > 1 for some *j*) or with two tails (*K*: *θ*_*j*_ ≠ 1 for some* j*). This paper addresses only the case of one-sided test; for the two-tail test the procedure is similar.

In the previous description it was assumed that the data (*x*_*j*_, *y*_*j*_) of each stratum* j* proceed from a double binomial distribution of sizes *m*_*j*_ and *n*_*j*_ and probabilities *p*_*j*_ and *q*_*j*_ in groups 1 and 2, respectively. Because in each stratum *j* there are two previously fixed values (*m*_*j*_ and *n*_*j*_) the model will be referred to as Model 2; the model is very frequently used in practice so that it will serve here as a basis for defining and illustrating the procedures MC and MCB. If in each stratum there is conditioning in the observed value *z*_*j*_ = *x*_*j*_ + *y*_*j*_, then one has Model 3; now the three values *m*_*j*_, *z*_*j*_, and *N*_*j*_ are previously fixed in each stratum *j* and the only variable *x*_*j*_ arises from a hypergeometric distribution. If only the values of *N*_*j*_ are fixed in each stratum *j*, one will get Model 1: $\left(x_{j},y_{j},{\overset{-}{x}}_{j}\right)$ proceeding from a multinomial distribution. Finally, if only the global sample size *N* is fixed (so that now even the values for *N*_*j*_ are obtained at random), one will have Model 0. With conditioning in the appropriate marginal, the model *X* leads to the model (*X* + 1). Therefore, whatever the initial model (i.e., whatever the sampling method for the data obtained), by conditioning in all the nonfixed marginals one always obtains Model 3 (which is the one covered by Birch and Mantel and Haenszel).

Each model produces a different sample space, which is formed by the set of all possible values of the set of variables involved in the same. For example, the sample space of stratum *j* under Model 2 consists of (*m*_*j*_ + 1)×(*n*_*j*_ + 1) possible values of (*x*_*j*_, *y*_*j*_). Each transition from a Model *X* to Model (*X* + 1) constitutes a loss of information, because the number of points of the new sample space is very much smaller than that of the previous one. Probably the most dramatic transition is that of Models 2 to 3, a transition in which the loss of information may reach 26% for *J* = 1 [5]. In addition, each transition implies using a conditional rather than an unconditional method of eliminating nuisance parameters, something which is generally never advisable [13].

The data in Table 2, which are given by Li et al. [12], are taken from preliminary analysis of an experiment of three groups to evaluate whether thymosin (treatment 1), compared to a placebo (treatment 2), has any effect on the treatment of bronchogenic carcinoma patients receiving radiotherapy. The one-sided *p* values are *P*_Birch_ = 0.1563 by global conditional stratified exact test and *P*_1_ = 0.80073, *P*_2_ = 0.57143, and *P*_3_ = 0.14706 by Fisher's individual conditional exact test in each stratum. If the global test is carried out to an error $\overset{-}{\alpha }=0.1563$ we conclude *K*, so that now *θ*_*j*_ > 1 at least once. However no individual test has significance if these are carried out to an alpha error that respects the former global error; for example, by using Bonferroni's method, the smaller of the three *p* values *P*_3_ = 0.14706 > 0.1563/3. The same thing occurs if asymptotic tests are used. Our aim is to define procedures in which these incompatibilities will not occur.

### 2.2. Conditional Tests Obtained by Using Classic Methods (Model 3)

The *p* value of exact test is *P*_Birch_ = 0.1563.  Table 3 shows this value and the remaining *p* values in this paper. This result is based on determining the probability of all the configurations (*x*_*j*_∣*N*_*j*_, *m*_*j*_, *z*_*j*_), *j* = 1,2,…, *J*, such as *S* = ∑_*j*_ *x*_*j*_ ≥ *S*_0_ = ∑_*j*_ *x*_*j*0_ = 27. Here *S* is a test statistic determining the order in which the points of the sample space (*x*_1_, *x*_2_, *x*_3_) enter the region *R*, a region whose probability under *H* yields the value of *P*_Birch_. Note that as the sample spaces in each stratum are 9 ≤ *x*_1_ ≤ 11, 8 ≤ *x*_2_ ≤ 9, and 5 ≤ *x*_3_ ≤ 8, the possible values of (*x*_1_, *x*_2_, *x*_3_) will be 3 × 2 × 4 = 24, which is the total number of points in the global sample space; of these, four belong to *R* (three with *S* = 27 and one with *S* = 28), so that 4/24 = 0.1667. Moreover note that, under the original Model 2, the number of points in the sample space of strata 1, 2, and 3 are (*m*_*j*_ + 1)×(*n*_*j*_ + 1) = (11 + 1)×(13 + 1), (9 + 1)×(12 + 1), and (8 + 1)×(10 + 1), respectively. The total points for the global sample space will be 168 × 130 × 99: more than two million, compared to only 24 in Model 3. To determine the value *P*_Birch_ have developed various programs (see references in [14]); an easy way to get it is through http://www.openepi.com/Menu/OE_Menu.htm (option “Two by Two Table”).

The asymptotic test of Mantel-Haenszel based on ∑*x*_*j*_ is asymptotically normal with mean ∑*E*_*j*_ = ∑_*j*_ *m*_*j*_ *z*_*j*_/*N*_*j*_ and variance $\sum_{}V_{j}=\sum_{}m_{j}n_{j}z_{j}{\overset{-}{z}}_{j}/N_{j}^{2}(N_{j}-1)$. Therefore the contrast statistic is *χ*_MH_ = (∑*x*_*j*_ − ∑*E*_*j*_)/(∑*V*_*j*_)^0.5^, whose *p* value *P*_MH_ = 0.0760 patently does not agree with *P*_Jung_ = 0.1563. However because the variable *S* is discrete, it is convenient to carry out a continuity correction [15]. As* S* jumps one space at a time, the cc should be 0.5 and so the statistic with cc will be *χ*_MHc_ = (∑*x*_*j*_ − ∑*E*_*j*_ − 0.5)/(∑*V*_*j*_)^0.5^ [8]. The new *p* value *P*_MHc_ = 0.1573 itself is already compatible with the exact value.

### 2.3. MC and MCB Tests Based on the Criterion of the Multiple Comparisons: General Observations

Let us suppose that in each stratum the hypotheses *H*_*j*_: *θ*_*j*_ = 1 versus *K*_*j*_: *θ*_*j*_ > 1 to error *α*_*j*_ are contrasted. Thereby *H* = ∩*H*_*j*_ and *K* = ∪*K*_*j*_. If the global null hypothesis *H* is rejected when there exists at least one *j* in which the individual test rejected *H*_*j*_, then the alpha error $\overset{-}{\alpha }$ of the global test (*H* versus *K*) will be [16]

(1) $$\begin{array}{c} \overset{-}{\alpha }=1-\underset{}{\prod }\left(\;1-\alpha_{j}\;\right). \end{array}$$

In particular, if *α*_*j*_ = *α*  (∀*j*) method MC is obtained (the “method of the multiple comparisons”), and its global alpha error will be

(2) $$\begin{array}{c} \overset{-}{\alpha }=1-{\left(\;1-\alpha \;\right)}^{J}. \end{array}$$

Method MC guarantees the compatibility of the results of the global test and of the individual tests, because the global test is significant if and only if at least one of the individual tests is so. When *J* = 1, the global test is the same as the individual test.

On the basis of the above, in general the test can be defined as follows. In each stratum *j* an order statistic *S*_*j*_ will have been defined which allows the *p* value for each one of its points to be determined. If the points from all strata are mixed, they are ordered from the lowest value of their *p* value to the highest and will be introduced one by one into the global critical region *R* until a given condition (stopping rule) has been verified; then *R* = ∪*R*_*j*_, with *R*_*j*_ the critical region formed by the points in the stratum *j* which belong to *R*. Let *α*_*j*_ be the largest of the *p* values of the points in *R*_*j*_. The real global alpha error ${\overset{-}{\alpha }}_{\text{MC}}$ of the test constructed thus will be given by expression (1).

When the stopping rule is “stop introducing points into *R* when the maximum of the *α*_*j*_ is as close as possible to *α* (but less than or equal to *α*),” with *α* given by

(3) $$\begin{array}{c} \alpha =1-\sqrt[{J}]{1-\overset{-}{\alpha }}, \end{array}$$

then method MC is obtained, and this method simultaneously controls global error $\overset{-}{\alpha }$ and the individual error *α*. Now, the critical region *R*_*j*_ = *R*_*j*MC_ of each stratum consists of all the points whose *p* value is smaller or equal to *α*, *α*_*j*_ = *α*_*j*MC_ ≤ *α*, *R* = *R*_MC_ = ∪*R*_*j*MC_ and the real global error will be ${\overset{-}{\alpha }}_{\text{MC}}=1-\prod_{}\left(1-\alpha_{j\text{MC}}\right)\leq 1-{\left(1-\alpha \right)}^{J}=\overset{-}{\alpha }$.

It is a simpler process to obtain the *p* value *P*_MC_ of some observed data. Let *P*_*j*_ be the *p*-value of the individual test in stratum *j*. The first individual alpha error for which *K* is concluded will be *α* = *P*_0_ = min⁡_*j*_ *P*_*j*_, so that for expression (2) the *p* value of the global text will be

(4) $$\begin{array}{c} P_{\text{MC}}=1-{\left(\;1-P_{0}\;\right)}^{J}. \end{array}$$

When the stopping rule is “stop introducing points into *R* when 1 − ∏(1 − *α*_*j*_) is the closest possible to $\overset{-}{\alpha }$ (but smaller than or equal to $\overset{-}{\alpha }$),” method MCB is obtained (the method “based on the multiple comparisons”). Because now only the global error $\overset{-}{\alpha }$ is controlled, its goal is similar to that of Jung's method [10]. The method MCB causes that *R*_*j*_ = *R*_*j*MCB_, *α*_*j*_ = *α*_*j*MCB_, *R* = *R*_MCB_ = ∪*R*_*j*MCB_ and the real global error is ${\overset{-}{\alpha }}_{\text{MCB}}=1-\prod_{}\left(1-\alpha_{j\text{MCB}}\right)\leq \overset{-}{\alpha }$. Note that *R*_MC_⊆*R*_MCB_, since ${\overset{-}{\alpha }}_{\text{MC}}\leq \overset{-}{\alpha }$, something to be expected given that method MC controls two errors and the MCB method controls only one of these.

Let us see how we can obtain the *p* value *P*_MCB_ of some observed data in which *P*_0_ = *P*_1_ for example. The region *R*_MCB_ which yields the first significance of the global test is obtained when the observed point in stratum 1 is the last introduced into *R*_MCB_, that is, when *α*_1MCB_ = *P*_0_; in the other strata it should be *α*_*j*MCB_ ≤ *P*_0_, but as close as possible to *P*_0_. Thus the *p* value will be *P*_MCB_ = 1 − ∏(1 − *α*_*j*MCB_). It can now be seen that *α*_*j*MCB_ = *α*_*j*MC_ where *α*_*j*MC_ are the values of the MC test when this is carried out to the error *α* = *P*_0_. Therefore *P*_MCB_ ≤ *P*_MC_ and, for effects of calculating the *p* value *P*_MCB_, the *p* values *α*_*j*MCB_ = *α*_*j*MC_ and the regions *R*_*j*MC_ = *R*_*j*MCB_ will be written just as *α*_*j*_^∗^ and *R*_*j*_^∗^, respectively. Thus, if *α*_*j*_^∗^ is the largest *p* value in stratum *j* which is smaller than or equal to *P*_0_,

(5) $$\begin{array}{c} P_{\text{MCB}}=1-\prod_{}\left(\;1-\alpha_{j}^{*}\;\right). \end{array}$$

Methods MC and MCB may be applied with exact methods or with asymptotic methods and to any of the three models, as illustrated in the following sections.

### 2.4. MC and MCB Tests under Model 3

The* p* values of the Fisher exact test in each stratum are *P*_1_ = 0.80073, *P*_2_ = 0.57143, and *P*_3_ = 0.14706. So, *P*_0_ = *P*_3_ = 0.14706 and *P*_MC_ = 0.3795 by expression (4). In order to apply method MCB the critical regions *R*_*j*_^∗^ (*j* = 1 and 2) must be determined to the objective error *α* = 0.14706 = *P*_0_ = *α*_3_^∗^. For *j* = 1, 9 ≤ *x*_1_ ≤ 11 with *Pr*⁡{*x*_1_ = 11∣*H*_1_} = 0.2862 > *P*_0_; thus *R*_1_^∗^ = *ϕ* and *α*_1_^∗^ = 0. This same occurs for *j* = 2 (*α*_2_^∗^ = 0). For expression (5), *P*_MCB_ = 0.1471 (smaller than *P*_Jung_). Generally speaking the critical region of Birch [9] and Jung [10] has the form *S* = ∑_*j*_ *x*_*j*_ ≥ *S*_0_ = ∑_*j*_ *x*_*j*0_, while that of method MCB is in the form ∪{*x*_*j*_ ≥ *x*_*j*_^∗^}, with *x*_*j*_^∗^ ≥ *x*_*j*0_. It can be proved that this generally implies that the Birch method will yield a* p* value smaller than or equal to that of method MCB when the* p* values *P*_*i*_ are similar or when the observed values *x*_*j*0_ are the highest possible.

Let us now apply an asymptotic test. In general, whatever the model is, the appropriate statistic is the chi-squared statistic [6]:

(6) $$\begin{array}{c} \chi_{j}=\frac{x_{j}{\overset{-}{y}}_{j}-y_{j}{\overset{-}{x}}_{j}-c_{j}}{\sqrt{m_{j}n_{j}z_{j}{\overset{-}{z}}_{j}/\left(N_{j}-1\right)}}. \end{array}$$

The appropriate value for the continuity correction *c*_*j*_ depends on the assumed model, and that value is what causes the results of the three models to be different. When *c*_*j*_ = 0  (∀*j*) Pearson's classic chi-squared statistic is obtained. In the case here of Model 3, by making *c*_*j*_ = *N*_*j*_/2 the classic statistic *χ*_3*j*_ (or the Yates chi-squared statistic) is obtained. Its maximum value is reached in stratum 3 (*χ*_33_ = 1.0308), which yields the* p* values *P*_0_ = 0.15132 and *P*_MC_ = 0.3887. In order to apply method MCB, one must obtain in the other two strata the first value *χ*_3*j*_^∗^ of *χ*_3*j*_ which is larger than or equal to *χ*_33_. As there is none, *α*_1_^∗^ = *α*_2_^∗^ = 0, *α*_3_^∗^ = 0.15132 and *P*_MCB_ = 0.1513. Note that the asymptotic* p* values are similar to the exact ones, both with method MC and with method MCB. Despite the small size of the samples, the asymptotic methods function well (something which also occurs with the rest of the methods, as will be seen).

### 2.5. MC and MCB Tests under Model 2

The data in the example in reality proceeds from Model 2. In determining the* p* value *P*_*j*_ of an observed table of Model 2 (*x*_*j*0_, *y*_*j*0_∣*m*_*j*_, *n*_*j*_) the same steps are followed as in Model 3 (except the last, which is special): (1) define an order statistic *S*_*j*_(*x*_*j*_, *y*_*j*_∣*m*_*j*_, *n*_*j*_), which does not need to be the same one in each stratum; (2) determine the set of points *R*_*j*_ = {(*x*_*j*_, *y*_*j*_∣*m*_*j*_, *n*_*j*_)∣*S*_*j*_(*x*_*j*_, *y*_*j*_∣*m*_*j*_, *n*_*j*_) ≥ *S*_*j*0_(*x*_*j*0_, *y*_*j*0_∣*m*_*j*_, *n*_*j*_)}; (3) calculate the probability of *R*_*j*_ under *H*_*j*_: *p*_*j*_ = *q*_*j*_ = *π*_*j*_ given by $\alpha_{j}\left(\pi_{j}\right)=\sum_{R_{j}}C_{m_{j},x_{j}}C_{n_{j},y_{j}}\pi_{j}^{z_{j}}{\left(1-\pi_{j}\right)}^{{\overset{-}{z}}_{j}}$; and (4) determine the* p* value as *P*_*j*_ = max⁡_*π*_*j*__⁡*α*_*j*_(*π*_*j*_), where *π*_*j*_ is the nuisance parameter that is eliminated by maximization (the most complicated step). Note that *π*_*j*_ is the marginal probability of columns under *H*_*j*_. In the case of Model 3 there is only one order statistic *S*_*j*_ possible [17], because the convexity of the region *R*_*j*_ must be verified and the points ordered “from the largest to the smallest value of *x*_*j*_.” In the case of Model 2 there are many possible test statistics. One of these is the order *F*_*j*_ of Boschloo [18]: order the points from the smaller to larger value of its one-tailed* p* value obtained using the Fisher exact test. It is already known [19] that the unconditional test based on the order *F*_*j*_ is uniformly more powerful (UMP) than Fisher's own exact test. Although no unconditional order is UMP compared to the rest, the generally most powerful order is [3] the complex statistic *B*_*j*_ of Barnard [20].

As far as we know, the only program that carries out the above calculations for the statistic *B*_*j*_ is SMP.EXE, which may be obtained free of charge at http://www.ugr.es/local/bioest/software.htm. The program also gives the solution for other simpler test statistics. Using this program, because the minimum* p* value is *P*_3_ = 0.05653 then *P*_MC_ = 0.1602. In order to obtain *P*_MCB_ one has to proceed as in the previous section, although now the process is now somewhat more difficult. In stratum 1, the table (*x*_1_, *y*_1_) = (11,10) is the one that gives a larger* p* value *α*_1_^∗^ = 0.05462, but smaller than or equal to *α*_3_^∗^ = 0.05653. In stratum 2 the results are (*x*_2_, *y*_2_) = (4,1) and *α*_2_^∗^ = 0.05069. So, *P*_MCB_ = 0.1533, a value which is similar to that of *P*_Birch_ (the results are alike if other order statistics of the program SMP.EXE are used). It can be seen that the use of the unconditional method allows the inherent conservatism in the definitions of methods MC and MCB to be reduced.

In order to carry out the asymptotic test we shall use the optimal version of expression (6) for Model 2: *χ*_2*j*_ is the value of expression (6) when *c*_*j*_ = 1 (or 2) if *m*_*j*_ ≠ *n*_*j*_ (or *m*_*j*_ = *n*_*j*_) [6]. Now the maximum value is *χ*_23_ = 1.5805, whereby *P*_3_ = 0.05700 and *P*_MC_ = 0.1614 (a value, i.e., very near the 0.1602 of the exact method). Proceeding as above, the first values *χ*_2*j*_^∗^ of *χ*_2*j*_ (*j* = 1 or 2) which are larger than or equal to *χ*_23_ are *χ*_21_^∗^ = 1.5822 for (*x*_1_, *y*_1_) = (10,8) and *χ*_22_^∗^ = 1.6056 for (*x*_2_, *y*_2_) = (2,0). This makes *α*_1_^∗^ = 0.05680, *α*_2_^∗^ = 0.05418, and *P*_MCB_ = 0.1588 (which is also a value, i.e., very close to the 0.1533 of the exact method).

### 2.6. MC and MCB Tests under Models 1 and 0

Let us suppose now that the data contained in the example in Table 2 proceed from Model 1. The determining of the* p* value *P*_*j*_ of an observed table $(x_{j0},y_{j0},{\overset{-}{y}}_{j0}|n_{j}$) is the same as in Model 2, but now the calculations are more complicated because the nuisance parameters must be eliminated (the marginal probabilities of rows and columns under *H*_*j*_). Again there are many possible test statistics [1, 21], although none of them is UMP compared to the others. The generally more powerful statistic is again Barnard's *B*_*j*_ statistic [22] and, as far as we know, the only program to apply it is TMP.EXE which may be obtained free of charge at http://www.ugr.es/local/bioest/software.htm. The program also gives the solution using other simpler test statistics. Using this program, the minimum* p* value is *P*_3_ = 0.04472 and from this *P*_MC_ = 0.1282 (substantially smaller than *P*_Birch_).

In order to carry out the asymptotic test we shall use the optimal version of expression (6) for Model 1: *χ*_1*j*_ is the value of expression (6) when *c*_*j*_ = 0.5  ∀*j* [6]. The statistic is given by Pirie and Hamdan [23]. Now the maximum value is *χ*_13_ = 1.6149, with the result that *P*_3_ = 0.05317 and *P*_MC_ = 0.1512.

Method MCB (which is very laborious to calculate) is omitted here, because the large number of points in the sample space will make *α*_1_^∗^ ≈ *α*_2_^∗^ ≈ *α*_3_^∗^ = *P*_3_ and so *P*_MC_ ≈ *P*_MCB_. Note that stratum 1 under Model 2 consists of (*m*_1_ + 1)(*n*_1_ + 1) = (11 + 1)×(13 + 1) = 168 points, but under Model 1 it consists of (*N*_1_ + 1)(*N*_1_ + 2)(*N*_1_ + 3)/6 = 25 × 26 × 27/6 = 2,925 points. For similar reasons, Model 0 can be treated as if it were Model 1 (by conditioning in the real obtained values *N*_*j*_).

## 3. Sample Size under Model 2

### 3.1. Example and Conditional Solutions Obtained by Classic Methods

Jung [10] proposes a sample size calculation for its stratified exact test. For the example described in Section 2.1, he accepts Model 2 and sets out a case study with *N*_*j*_ = *N*/3 and *m*_*j*_ = *N*/6. The aim is to determine the value of *N* for the alternative hypotheses (*θ*_1_, *θ*_2_, *θ*_3_) = (1,30,30), a type I error of $\overset{-}{\alpha }=0.1$ and a power of $1-\overset{-}{\beta }=0.8$. Jung also assumes that (*q*_1_, *q*_2_, *q*_3_) = (0.9,0.75,0.6), so that under the alternative hypothesis $p_{j}=\theta_{j}q_{j}/({\overset{-}{q}}_{j}+\theta_{j}q_{j})$. His solution is *N*_Jung_ = 62. From what can be deduced from other parts of his paper, the detailed solution is *n*_1_ = *n*_2_ = 20, *n*_3_ = 22, *m*_1_ = *m*_2_ = 10, and *m*_3_ = 11. These values are included in Table 4 (as well as the most relevant ones obtained in all the following). This sample size provides a real error of ${\overset{-}{\alpha }}_{\text{Jung}}=0.0565$ and a real power of $1-{\overset{-}{\beta }}_{\text{Jung}}=0.8105$.

Let us suppose that generally *n*_*j*_ = *k*_*j*_ *m*_*j*_, with *k*_*j*_ known values, and that the aim is to determine the values *m*_*j*_ which guarantee the desired power, which implies using Model 2. The reasoning that follows is the same as that with which Casagrande et al. [24] and Fleiss et al. [25] obtained the classic formula for sample size in the comparison of two independent proportions. The solutions without cc that follow are a special case of those of Jung et al. [11]. The test *χ*_MHc_ in Section 2.2 is based on the statistic $\sum_{}\left(x_{j}-E_{j}\right)-0.5=\sum_{}{\hat{D}}_{j}-0.5$, where ${\hat{D}}_{j}=(k_{j}x_{j}-y_{j})/(k_{j}+1)$. Because ${\hat{D}}_{j}$ is distributed asymptotically as a normal distribution with the mean *D*_*j*_ = *k*_*j*_ *m*_*j*_(*p*_*j*_ − *q*_*j*_)/(*k*_*j*_ + 1) and the variance $S_{j}^{2}=k_{j}m_{j}(k_{j}p_{j}{\overset{-}{p}}_{j}+q_{j}{\overset{-}{q}}_{j})/{\left(k_{j}+1\right)}^{2}$, $\hat{D}=\sum_{}{\hat{D}}_{j}$ will be asymptotically normal with the mean *D* = ∑*D*_*j*_ and the variance *S* ^2^ = ∑*S*_*j*_^2^. Under *H*, *p*_*j*_ = *q*_*j*_ = *π*_*j*_  (∀*j*), with the result that the mean and variance of $\hat{D}$ will be *D*_*H*_ = 0 and of $S_{H}^{2}=\sum_{}k_{j}m_{j}\pi_{j}{\overset{-}{\pi }}_{j}/(k_{j}+1)$, respectively, with ${\overset{-}{\pi }}_{j}=1-\pi$_*j*_. Because under *H* the nuisance parameter *π*_*j*_ is estimated by *z*_*j*_/*N*_*j*_, it is usual to substitute it by its average value under *K*, that is, by *π*_*j*_ = (*p*_*j*_ + *k*_*j*_ *q*_*j*_)/(*k*_*j*_ + 1); hence ${\overset{-}{\pi }}_{j}=({\overset{-}{p}}_{j}+k_{j}{\overset{-}{q}}_{j})/(k_{j}+1)$. Consequently the statistic $\hat{D}$ will reach significance in the critical value *D* ^∗^ which verifies $\overset{-}{\alpha }=\mathrm{Pr}\{\hat{D}\geq D^{*}|H\}=\mathrm{Pr}\{z\geq (D^{*}-0.5)/S_{H}\}$, in which the number 0.5 corresponds to the cc indicated above and *z* refers to a normal standard variable. Therefore $D^{*}=z_{1-\overset{-}{\alpha }}S_{H}+0.5$, with $z_{1-\overset{-}{\alpha }}$ the $100\times (1-\overset{-}{\alpha })$-percentile of the normal standard distribution. Under *K* the parameters *D* = *D*_*K*_ and *S* ^2^ = *S*_*K*_^2^ are obtained in the values *p*_*j*_ and *q*_*j*_ which specify *K*: *D*_*K*_ = ∑*k*_*j*_ *m*_*j*_(*p*_*j*_ − *q*_*j*_)/(*k*_*j*_ + 1) and $S_{K}^{2}=\sum_{}k_{j}m_{j}(k_{j}p_{j}{\overset{-}{p}}_{j}+q_{j}{\overset{-}{q}}_{j})/{\left(k_{j}+1\right)}^{2}$. Given the above, the error beta will be

(7) β−MHcPr⁡D^≤D∗ ∣ K=Pr⁡z≤z1−α−SH+1−DKSK.

If the solution is restricted to the case of *m*_*j*_ = *m*  (∀*j*), by making $-z_{1-\overset{-}{\beta }}$ equal to the fraction of expression (7) and by working out *m*, one obtains the equation $m\delta -m^{0.5}[z_{1-\overset{-}{\alpha }}\sigma_{0}+z_{1-\overset{-}{\beta }}\sigma_{1}]-0.5=0$, where *δ* = ∑*k*_*j*_(*p*_*j*_ − *q*_*j*_)/(*k*_*j*_ + 1), $\sigma_{0}^{2}=\sum_{}k_{j}\pi_{j}{\overset{-}{\pi }}_{j}/(k_{j}+1)$, and $\sigma_{1}^{2}=\sum_{}k_{j}(k_{j}p_{j}{\overset{-}{p}}_{j}+q_{j}{\overset{-}{q}}_{j})/{\left(k_{j}+1\right)}^{2}$; therefore

(8) m=m041+1+2m0δ2where  m0=z1−α−σ0+z1−β−σ1δ2.

The solutions *m*_0_ and *m* are those of the tests *χ*_MH_ and *χ*_MHc_, respectively. Frequently *k*_*j*_ = 1  (∀*j*); in this case expression (8) explicitly takes the following form:

(9) m=m041+1+4m0∑pj−qj2with  m0=z1−α−∑pj+qjp−j+q−j/2+z1−β−∑pjp−j+qjq−j∑pj−qj2.

For the example at the beginning of this section (in which *k*_*j*_ = 1), if at first we restrict the solution to *m*_1_ = *m*_2_ = *m*_3_ = *m*, expression (9) indicates that *m*_0_ = 8.27 and *m* = 11.3. Assuming that in this example the values of *m*_*j*_ are allowed to differ at most by 1, then the solution that is sought must be 8 ≤ *m*_*j*_ ≤ 9  (∀*j*) without cc or 11 ≤ *m*_*j*_ ≤ 12  (∀*j*) with cc. In the second phase, expression (7) indicates that in *m*_1_ = *m*_2_ = 11 and *m*_3_ = 12 is the first time that ${\overset{-}{\beta }}_{\text{MHc}}$ (=0.183) ≤ 0.2, so that this is the solution with cc that was being sought (*N* = 68). The solution without cc is obtained in the same way (*m*_1_ = *m*_2_ = 8, *m*_3_ = 9, and *N* = 50), but it is too liberal.

### 3.2. Solution Using the Exact Method MC

For fixed values of the global error $\overset{-}{\alpha }$ and the sample sizes (*m*_*j*_, *n*_*j*_), the method MC described in Section 2.3 allows one to obtain the critical region *R*_*j*MC_ and the real type 1 error ${\overset{-}{\alpha }}_{\text{MC}}\leq \overset{-}{\alpha }$. Moreover, let *β*_*j*MC_ be the error beta for each individual test, with 1 − *β*_*j*MC_ equal to the probability of the region *β*_*j*MC_ under *K*_*j*_. Because of the way method MC was defined, the real global error beta will be

(10) β−MC∏jβjMC=∏j=1J1−∑RjMCmjxjnjyjpjxjp−jx−jqjyjq−jy−j.

If ${\overset{-}{\beta }}_{\text{MC}}\leq \overset{-}{\beta }$, these values {(*m*_*j*_, *n*_*j*_)} guarantee the desired power. If ${\overset{-}{\beta }}_{\text{MC}}>\overset{-}{\beta }$, it is necessary to increase some values of *m*_*j*_ and/or *n*_*j*_ and to repeat the previous procedure.

Let us initially assume that *m*_*j*_ = *n*_*j*_. The process for determining the sample sizes *m*_*j*_ may be shortened if it begins with a value *m*_*j*_ = *m*  (∀*j*) like that of expression (8). With the method MC, one obtains that *m* = 12 is not a solution because ${\overset{-}{\beta }}_{\text{MC}}=0.2262>0.2$, but *m* = 13 is a solution because ${\overset{-}{\beta }}_{\text{MC}}=0.1723\leq 0.2$. The solution can now be refined allowing values *m*_*j*_ to differ by a maximum of one. The final solution is *m*_1_ = *m*_2_ = 12, *m*_3_ = 13 (*N* = 74), ${\overset{-}{\alpha }}_{\text{MC}}=0.0881$, and ${\overset{-}{\beta }}_{\text{MC}}=0.1880$.

Unconditioned tests are more powerful when the sample sizes are slightly different [3], since the number of ties that produces any statistic *S*_*j*_ that is used is reduced. By planning *n*_*j*_ = *m*_*j*_ + 1 and making the values of *m*_*j*_ consecutive, the solution *m*_1_ = 10, *m*_2_ = 11, and *m*_3_ = 12 (*N* = 69) is obtained, with ${\overset{-}{\alpha }}_{\text{MC}}^{}=0.0924$ and ${\overset{-}{\beta }}_{\text{MC}}^{}=0.1821$ (the solution based on *n*_*j*_ = *m*_*j*_ − 1 is worse). Actually, stratum 1 is of virtually no interest since in it *H*_1_ = *K*_1_. Despite everything, if it is introduced, the configuration *n*_*j*_ = *m*_*j*_ + 1, *m*_1_ = 1, *m*_2_ = 11 and *m*_3_ = 12 (*N* = 51) is correct because ${\overset{-}{\alpha }}_{\text{MC}}^{}=0.0602$ and ${\overset{-}{\beta }}_{\text{MC}}^{}=0.1833$.

### 3.3. Solution Using the Asymptotic Method MC Based on the Chi-Square Test with cc

In the following the procedure is the same as in Section 2.1, assuming for the moment that *m*_*j*_ and *n*_*j*_ can be any values. The numerator of *χ*_2*j*_ may be written as ${\hat{d}}_{j}-c_{j}$, where *c*_*j*_ is the cc of Model 2 (*c*_*j*_ = 2 or 1 depending on whether *m*_*j*_ and *n*_*j*_ are equal or different, resp.) and ${\hat{d}}_{j}=n_{j}x_{j}-m_{j}y_{j}$ (the base statistic for the test) is asymptotically normal with mean *d*_*j*_ = *m*_*j*_ *n*_*j*_(*p*_*j*_ − *q*_*j*_) and variance $s_{j}^{2}=m_{j}n_{j}(n_{j}p_{j}{\overset{-}{p}}_{j}+m_{j}q_{j}{\overset{-}{q}}_{j}$).

Under *H*_*j*_, *p*_*j*_ = *q*_*j*_ = *π*_*j*_ and ${\hat{d}}_{j}$ is asymptotically normal with mean 0 and variance $s_{Hj}^{2}=N_{j}m_{j}n_{j}\pi_{j}{\overset{-}{\pi }}_{j}$, with ${\overset{-}{\pi }}_{j}=1-\pi_{j}$. Because under *H*_*j*_ the nuisance parameter *π*_*j*_ is estimated by *z*_*j*_/*N*_*j*_, it is usual to substitute it by its average value under *K*_*j*_, that is, by *π*_*j*_ = (*m*_*j*_ *p*_*j*_ + *n*_*j*_ *q*_*j*_)/*N*_*j*_; hence ${\overset{-}{\pi }}_{j}=(m_{j}{\overset{-}{p}}_{j}+n_{j}{\overset{-}{q}}_{j})/N_{j}$. If each individual test is realized to the error *α* of expression (3), the critical value *d*_*j*_^∗^ for ${\hat{d}}_{j}$ will verify $\alpha =\mathrm{Pr}\{{\hat{d}}_{j}\geq d_{j}^{*}|H_{j}\}=\mathrm{Pr}\{z\geq (d_{j}^{*}-c_{j})/s_{Hj}\}$, in which the value *c*_*j*_ corresponds to the cc indicated above; therefore *d*_*j*_^∗^ = *z*_1−*α*_ *s*_*Hj*_ + *c*_*j*_.

Under *K*_*j*_, ${\hat{d}}_{j}$ is asymptotically normal with mean *d*_*Kj*_ = *m*_*j*_ *n*_*j*_(*p*_*j*_ − *q*_*j*_) and variance $s_{Kj}^{2}=m_{j}n_{j}\left(n_{j}p_{j}{\overset{-}{p}}_{j}+m_{j}q_{j}{\overset{-}{q}}_{j}\right)$. Thus $\beta_{j}=\mathrm{Pr}\{{\hat{d}}_{j}\leq d_{j}^{*}|K_{j}\}=\mathrm{Pr}\{z\leq (z_{1-\alpha }s_{Hj}+c_{j}-d_{Kj})/s_{Kj}\}$ and applying the first equality in expression (10)

(11) $$\begin{array}{c} {\overset{-}{\beta }}_{\text{MC}}^{}=\underset{j}{\prod }\beta_{j\text{MC}}=\underset{j}{\prod }\mathrm{Pr}\left\{\;z\leq \frac{z_{1-\alpha }s_{Hj}+c_{j}-d_{Kj}}{s_{Kj}}\;\right\}, \end{array}$$

in particular, if *m*_*j*_ = *n*_*j*_ = *m*  (∀*j*), then *c*_*j*_ = 2, and

(12) β−MC=∏jPr⁡z≤z1−αmmpj+qjp−j+q−j/2+2−m2pj−qjmmpjp−j+qjq−j.

For the data in the example, *α* = 1 − 0.9^1/3^ = 0.03451 and by making *m*_*j*_ = *m*  (∀*j*) the solution, the solution based on expression (12) is *m* = 12. This solution can be refined by allowing the values of *m*_*j*_ to differ by a maximum of one, in which case the new solution, now based on expression (11), is *m*_1_ = 11, *m*_2_ = *m*_3_ = 12 (*N* = 70) with ${\overset{-}{\beta }}_{\text{MC}}^{}=0.1901$. If a cc is not carried out the solution is too liberal: *m*_1_ = *m*_2_ = 10, *m*_1_ = 11 (*N* = 62) with ${\overset{-}{\beta }}_{\text{MC}}^{}=0.1984$. By planning *n*_*j*_ = *m*_*j*_ + 1 and making the values of *m*_*j*_ consecutive, the solution *m*_1_ = 10, *m*_2_ = 11, and *m*_3_ = 12 is obtained (as in the exact method), with ${\overset{-}{\alpha }}_{\text{MC}}^{}$= ${\overset{-}{\beta }}_{\text{MC}}^{}=0.1759$. This is the same result as for the configuration at the end of the previous section.

## Figures and Tables

**Table 1:** Frequency data of 2 × 2 table for stratum *j*.

Treatment	Response		Total
	Yes	No	
1	*x*_*j*_	${\overset{-}{x}}_{j}$	*m*_*j*_
2	*y*_*j*_	${\overset{-}{y}}_{j}$	*n*_*j*_

Total	*z*_*j*_	${\overset{-}{z}}_{j}$	*N*_*j*_

**Table 2:** Response to thymosin in cancer patients (yes = success, no = failure).

	Stratum 1		Total	Stratum 2		Total	Stratum 3		Total
	Yes	No		Yes	No		Yes	No	
Thymosin	10	1	11	9	0	9	8	0	8
Placebo	12	1	13	11	1	12	7	3	10

Total	22	2	24	20	1	21	15	3	18

**Table 3:** *p* values obtained by various methods for the data in the example of Li et al. [12]. Each asymptotic method is placed directly below the exact method from which it proceeds.

Model	Test	Procedure	Statistic used	p value
3	Exact	Birch	Sum of successes (treated group)	0.1563
	Asymptotic	MH	*χ*_MH_ of Mantel-Haenszel (without cc)	0.0760
			*χ*_MH_ of Mantel-Haenszel (with cc)	0.1573
	Exact	MC	*p* value Fisher	0.3795
	Asymptotic		*χ*_3_ of Yates	0.3887
	Exact	MCB	*p* value Fisher	0.1471
	Asymptotic		*χ*_3_ of Yates	0.1513

2	Exact	MC	*p* value Barnard	0.1602
	Asymptotic		*χ*_2_ of Martín et al.	0.1614
	Exact	MCB	*p* value Barnard	0.1533
	Asymptotic		*χ*_2_ of Martín et al.	0.1588

1	Exact	MC	*p* value Barnard	0.1282
	Asymptotic		*χ*_1_ of Pirie and Hamdan	0.1512

Note: MH = Mantel-Haenszel test; MC = multiple comparisons method; MCB = method based on the multiple comparisons.

**Table 4:** Sample sizes by stratum (*m*_*j*_ = *n*_*j*_) and global (*N*) obtained by various methods for the data of Jung's example [10] under Model 2. Each asymptotic method is placed immediately below the exact method from which it proceeds.

Model	Test	Procedure	Stratum			*N*
			1	2	3	
Conditional	Exact	Jung	10, 10	10, 10	11, 11	62
	Asymptotic	*χ*_MH_ without cc	8, 8	8, 8	9, 9	50
		*χ*_MH_ with cc	11, 11	11, 11	12, 12	68

Unconditional	Exact	MC (Barnard's order)	12, 12	12, 12	13, 13	74
			10, 11	11, 12	12, 13	69
			1, 2	11, 12	12, 13	51
	Asymptotic	MC (*χ*_2_ with cc)	11, 11	12, 12	12, 12	70
			10, 11	11, 12	12, 13	69
			1, 2	11, 12	12, 13	51

Note: *χ*_MH_: *χ* of Mantel-Haenszel; MC = multiple comparisons method; *χ*_2_ = *χ* of Model 2.
